# Optical fiber ultrasound transmitter with electrospun carbon nanotube-polymer composite

**DOI:** 10.1063/1.4984838

**Published:** 2017-06-01

**Authors:** Radhika K. Poduval, Sacha Noimark, Richard J. Colchester, Thomas J. Macdonald, Ivan P. Parkin, Adrien E. Desjardins, Ioannis Papakonstantinou

**Affiliations:** 1 Department of Electronic and Electrical Engineering, University College London, London WC1E 7JE, United Kingdom; 2 Department of Medical Physics and Biomedical Engineering, University College London, London WC1E 6BT, United Kingdom; 3 Materials Chemistry Centre, Department of Chemistry, University College London, London WC1H 0AJ, United Kingdom

## Abstract

All-optical ultrasound transducers are promising for imaging applications in minimally invasive surgery. In these devices, ultrasound is transmitted and received through laser modulation, and they can be readily miniaturized using optical fibers for light delivery. Here, we report optical ultrasound transmitters fabricated by electrospinning an absorbing polymer composite directly onto the end-face of optical fibers. The composite coating consisting of an aqueous dispersion of multi-walled carbon nanotubes (MWCNTs) in polyvinyl alcohol was directly electrospun onto the cleaved surface of a multimode optical fiber and subsequently dip-coated with polydimethylsiloxane (PDMS). This formed a uniform nanofibrous absorbing mesh over the optical fiber end-face wherein the constituent MWCNTs were aligned preferentially along individual nanofibers. Infiltration of the PDMS through this nanofibrous mesh onto the underlying substrate was observed and the resulting composites exhibited high optical absorption (>97%). Thickness control from 2.3 *μ*m to 41.4 *μ*m was obtained by varying the electrospinning time. Under laser excitation with 11 *μ*J pulse energy, ultrasound pressures of 1.59 MPa were achieved at 1.5 mm from the coatings. On comparing the electrospun ultrasound transmitters with a dip-coated reference fabricated using the same constituent materials and possessing identical optical absorption, a five-fold increase in the generated pressure and wider bandwidth was observed. The electrospun transmitters exhibited high optical absorption, good elastomer infiltration, and ultrasound generation capability in the range of pressures used for clinical pulse-echo imaging. All-optical ultrasound probes with such transmitters fabricated by electrospinning could be well-suited for incorporation into catheters and needles for diagnostics and therapeutic applications.

All-optical ultrasound is an emerging tool for minimally invasive medical procedures, with possible applications in transseptal puncture, placenta imaging, and intravascular imaging.^1,2^ Ultrasound can be generated in coatings on an optical fiber tip through the photoacoustic effect by pulsed or modulated laser illumination.^2–6^ This principle is used in optical ultrasound transmitters which yield broad bandwidths, high pressures, high sensitivity, and electromagnetic noise immunity.^7^ When paired with fiber optic ultrasound receivers, for instance Fabry Pérot fiber hydrophones,^8^ miniature all-optical ultrasound probes can be realized for pulse-echo ultrasound imaging.

Coating materials possessing both high optical absorption and high coefficient of thermal expansion are desirable in order to achieve efficient optical ultrasound generation.^7,9^ Optical absorbers like metal nanoparticles and carbonaceous composites including carbon black, carbonized polymers, and carbon nanotubes (CNTs) have previously been considered.^2,4,6,10–13^ Composites of CNTs in polydimethylsiloxane (PDMS) elastomeric hosts are promising for ultrasound pressure generation in the clinical range (0.1–10's of MPa).^1,2,11,14^

Harnessing innovations in micro- and nanofabrication techniques can enhance the performance of optical ultrasound transmitters. A variety of methods have been reported for optical ultrasound transmitter fabrication. These include techniques such as dip-coating,^2,4^ spin-coating,^15^ chemical vapor deposition,^11,14,16^ metal evaporation,^6^ and etching.^12,17^ Dip-coated and spin-coated absorber-elastomer composites on glass substrates have been implemented for fabrication of carbonaceous optical ultrasound generating surfaces.^1,2,16^ Electrospun carbonized polyacrylonitrile nanofibers deposited on macroscale substrates (a flat glass slide and a concave lens) and then spin-coated with PDMS were also reported for laser ultrasound generation.^15^ In this instance, the area of the coated substrate was significantly larger than an optical fiber cross-section. Whilst ultrasound generating coatings can be deposited on optical fiber end-face by composite dip-coating,^1,2^ control of the absorber layer coating thickness can be challenging. Additionally, it is desirable to achieve high PDMS elastomer infiltration into the optical absorber, i.e., multi-walled carbon nanotubes (MWCNTs), to enhance thermoelastic heat transfer between the nanotubes and PDMS.^7^

In this study, we combine direct electrospinning of an optical absorber material on an optical fiber tip, with elastomer dip-coating, to form miniature optical ultrasound transmitters. Previously, electrospinning has only been reported on the curved side-surface of an optical fiber for the fabrication of a humidity sensor,^18^ but here we demonstrate electrospinning of a polymer composite directly onto an optical fiber end-face and highlight its capability for optical ultrasound generation. In our electrospun coating method, we use aqueous dispersions of MWCNTs, which can be functionalized by sonication with a commercially available ligand to achieve suitable aqueous MWCNT concentrations for formation of an optically absorbing MWCNT-polymer composite. This process is less complex than functionalization strategies necessary to disperse MWCNTs in organic solvents such as xylene which are compatible with polymers for optical ultrasound generation.^1,2^

Nanofibers produced by electrospinning possess high porosity, which results in surface areas several orders of magnitude higher than regular films.^19^ The porous optically absorbing planar mesh structure can assist elastomer (PDMS) infiltration and the high surface area can function to promote heat transfer between the absorber and elastomer constituents, beneficial for optical ultrasound generation. Electrospinning can also offer increased control over the coating thickness through variation of electrospinning time. Moreover, we postulate that the electrospun deposition can promote preferential in-plane alignment of MWCNTs along the optical fiber end-face. This could help enhance optical absorption efficiency in electrospun coatings relative to random MWCNT alignment, due to the optical absorption anisotropy of MWCNTs.^20,21^ It has been demonstrated that the co-polarized absorption cross-section of MWCNTs is larger by a factor of two compared to the cross-polarized absorption cross-section.^22^ Concurrently, since the light emerging from a multimode optical fiber cleaved at normal incidence is polarized in the plane parallel to its cleaved surface,^23^ MWCNT-based coatings with the nanotube axes oriented parallel to the optical fiber end-face could maximize optical absorption efficiency. Furthermore, to better understand the generation of ultrasound in coatings made with this technique, the electrospun transmitters were compared with an equivalent dip-coated reference transmitter in which the same constituent materials were used to form a solid film coating, devoid of the electrospun nanostructure at the optical fiber tip.

A nanofibrous thermoelastic composite with MWCNTs, polyvinyl alcohol (PVA), and PDMS was developed for coating the end-face of optical fibers. MWCNTs with dimensions of 6–9 nm × 5 *μ*m (724769, Sigma Aldrich, UK) were functionalized for dispersion in deionized water (30 ml water) using cetyltrimethylammonium bromide (CTAB, 0.3 g) through a modification of the method described by Vilčáková *et al.*^24^ This solution was sonicated (Branson B-Series 1510, 100% power) for a minimum of 2 h to disperse the MWCNTs in water. Solutions with increasing concentrations of MWCNTs were considered with the aim of obtaining both high concentration and uniform electrospun fiber morphology with minimal nanoparticle clumping. Optimizing the nanotube loading in the nanofibrous materials has been shown previously to be an important factor in developing high performance electrospun composite materials.^25^ The sonicated MWCNT dispersion was left overnight to allow for settling of any stray non-functionalized MWCNTs or impurities. The dispersion containing 7.5 mg/ml of MWCNTs in water was the nanotube solution with the highest concentration that produced uniform continuous electrospun absorber nanofibers without agglomerations and was used in the fabrication of our ultrasound transmitters.

The precursor solution for electrospinning was prepared by pipetting out requisite volume of the MWCNT dispersion and heating it to 80 °C. To this heated dispersion, 9 wt. % of polyvinyl alcohol (PVA) (Sigma Aldrich 321584-25G, molecular wt. 89 000–98 000, 99% hydrolyzed) was added and stirred for 24 h on a heating plate at 70 °C. This precursor polymer composite of MWCNT and PVA solution was degassed prior to electrospinning.

Electrospun deposition of absorbing nanofibers was carried out directly onto an optical fiber end-face using a collector plate modified for substrate insertion (Fig. 1). A flat plate covered with aluminum foil (10 cm × 10 cm) and a central circular aperture (0.6 cm diameter) for optical fiber placement acted as the collector/grounding electrode for electrospinning. The end-face of a step-index, silica-core, silica-cladding multimode optical fiber (FG200LEA, Thorlabs, UK, numerical aperture 0.22) with core/cladding diameters of 200/220 *μ*m and cleaved at normal incidence was used as the substrate. The optical fiber end-face was aligned parallel to the collector plate surface with its distal end protruding by ∼1 mm through the aperture in the central region of the grounding plate. The proximal end of the optical fiber, along with the excess overhanging section, was supported behind the collector plate on a horizontal non-conducting plastic stand. On securing the substrate, the PVA-MWCNT composite precursor solution was electrospun at an ambient temperature of 21 °C and humidity between 50% and 60%. The solution was dispensed through a blunted 23 G needle at an applied potential difference of 9 kV, a flow rate of 0.5 ml/h, and a needle-to-collector separation of ∼12 cm. Following this, the optical fiber end-face was gently pushed out through the collector plate, resulting in optical fiber with electrospun nanofibers deposited on its end-face [Fig. 2(b)].

**FIG. 1.**
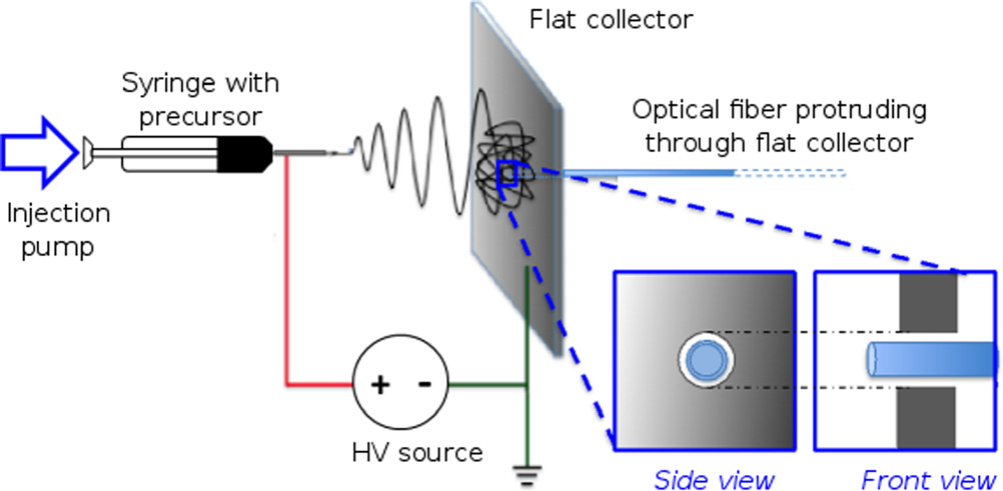
Schematic of the setup used for electrospun deposition of multiwalled carbon nanotube (MWCNT)—polyvinyl alcohol (PVA) optical absorber composite onto the optical fiber end-face. The optical fiber protruded through a hole in the flat collector (insets: side view and front view). The MWCNT-PVA precursor was deposited via a syringe and injection pump, through a blunt metallic needle that was held at a high voltage (HV) relative to the flat collector.

**FIG. 2.**
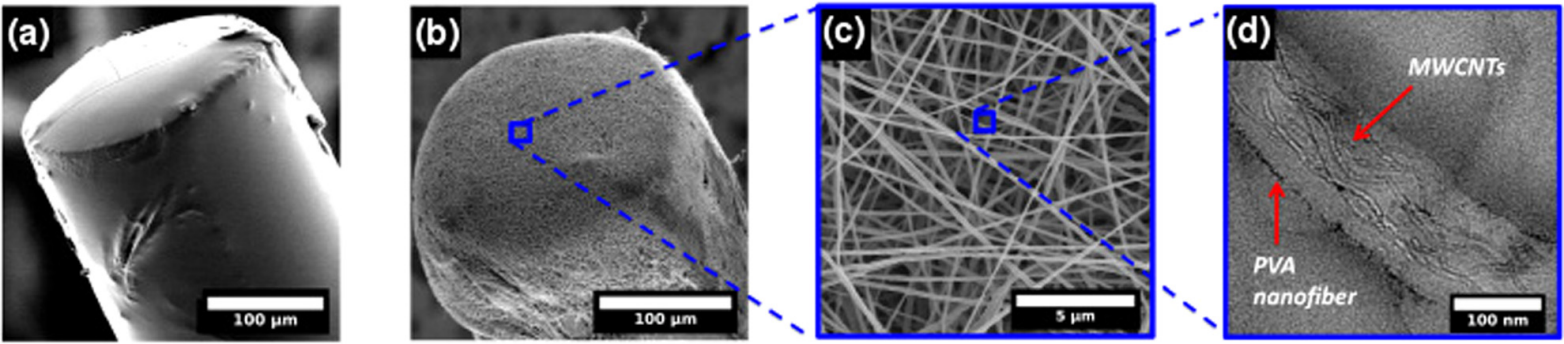
(a) SEM of the optical fiber ultrasound transmitter composed of a multiwalled carbon nanotube (MWCNT)—polyvinyl alcohol (PVA) composite following polydimethylsiloxane (PDMS) elastomer dip-coating. A smooth coating can be observed over the core area of the multimode optical fiber end-face. (b) SEM of the electrospun absorber coating on the optical fiber end-face, illustrating nanofibrous absorber mat deposition. (c) An expanded view of the SEM in (b), which shows electrospun MWCNT-PVA nanofibers (planar mesh structure) coated on the optical fiber end-face. (d) A TEM of the electrospun absorber nanofibers illustrating preferential alignment of the MWCNTs within the PVA nanofibers.

PDMS elastomer was incorporated by dip-coating the electrospun PVA-MWCNT coated optical fiber end-face to provide a high thermoelastic coefficient. Diluted PDMS (Sylgard 184, Dow Corning thinned with 30 wt. % toluene) was used to lower the viscosity of the elastomer solution to promote infiltration into the pores of the electrospun absorber. The dip-coated optical fiber end-face was left to cure facing up for at least 24 h at room temperature.

Optical absorption values were measured in an integrating sphere setup (FOIS-1, Ocean Optics, USA).^2^ Laser generated ultrasound pressure measurements were conducted as described in Colchester *et al.*^4^ The excitation light was provided by a Q-switched Nd:YAG 1064 nm laser (SPOT-10–500–1064, Elforlight, UK), with a pulse width of 2 ns, a repetition rate of 100 Hz, and a laser pulse energy of 11 *μ*J (fluence: 35 mJ/cm^2^). For pressure measurements, the optical fiber ultrasound transmitters were positioned in water facing a calibrated 75 *μ*m diameter needle hydrophone (SN 493, Precision Acoustics, UK) with a calibration range of 1–30 MHz and 1.5 mm separation between the transmitter and the hydrophone surface.

The fiber optic ultrasound transmitter with the electrospun absorber and dip-coated elastomer was imaged by scanning electron microscopy (SEM) and transmission electron microscopy (TEM) [Figs. 2(a)–2(d)]. SEM was performed using secondary electron imaging on a field emission instrument (JSM-6301F, JEOL, Japan) with an acceleration voltage of 5 kV, while TEM micrographs were collected using a JEOL 2010 TEM operating at 200 kV. The SEM image in Fig. 2(a) shows the uniform deposition of the composite coating at the end-face of a multimode optical fiber cleaved at normal incidence. Consequently, the resultant acoustic source has a diameter of 200 *μ*m, i.e., the diameter of the optical fiber core. Coverage of the optical fiber end-face with the planar nanofibrous MWCNT-PVA absorber mesh prior to PDMS dip coating is illustrated in Fig. 2(b), with the nanofiber layers largely parallel to the optical fiber end-face. The electrospun nanofiber diameters [Fig. 2(c)] were between 175 and 250 nm, with a mean diameter of 207 nm. Furthermore, the MWCNTs that were dispersed with random orientation in the polymer precursor are seen to be preferentially oriented along the electrospun nanofiber axis, under TEM [Fig. 2(d)]. This can be attributed to controlled polymer flow, in conjunction with the externally applied electrostatic field promoting orientation of MWCNTs along the length of the PVA nanofibers during electrospinning.^26^ Electrospinning resulted in orientation of the absorber MWCNT-PVA composite as stacked planar meshes on the optical fiber end-face [Fig. 2(b)], with an absence of uniaxial alignment on a macro scale. Since the optical pulse leaving the multimode optical fiber is plane polarized, planar alignment of MWCNT's axes within the planar nanofibrous absorber mesh should present an improvement in terms of optical absorption efficiency over random 3D arrangement of carbon nanotubes (for instance, as achieved with dip-coating).

The electrospinning process allowed for control of the coating thickness. With all other parameters kept constant, increasing the electrospinning time from 2 to 14 min correspondingly increased the electrospun coating thickness from 2.3 *μ*m to 41.4 *μ*m [Fig. 3(a)]. Moreover, complete elastomer infiltration into the electrospun absorber mesh was achieved [Fig. 3(b)]. Coating thickness and elastomer infiltration were ascertained through cross-sectional SEM, by replicating our coating process with a glass coverslip as the substrate, which was then fractured cryogenically for cross-sectional imaging.

**Figure:**
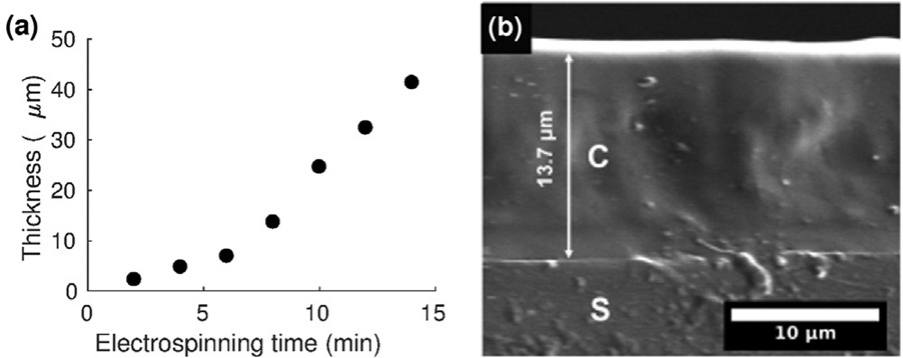
FIG. 3. (a) Variation of the electrospun composite thickness with electrospinning time. (b) Cross-sectional SEM of the composite [C] (thickness: 13.7 *μ*m; electrospinning time: 8 min), which demonstrates complete polydimethylsiloxane (PDMS) infiltration through the nanofibrous mat to the underlying glass substrate [S].

The increased coating thickness led to an increase in optical absorption and a change in the generated ultrasound pressure. A monotonic increase in optical absorption approaching unity was observed with increasing electrospinning time [Fig. 4(a-i)], as expected from the Beer-Lambert law, and the increased coating thickness. In contrast, the measured ultrasound peak-to-peak pressure reached a maximum of 1.59 MPa at an optimal electrospinning time of 8 min that corresponded to a coating thickness of 13.7 *μ*m and then dropped with a further increase in electrospinning time [Fig. 4(a-ii)]. This indicates that beyond a certain optimal coating thickness, there is a tradeoff between the increased optical absorption and simultaneous ultrasound attenuation (due to absorption and scattering of the generated ultrasound within the composite coating).^7,27^ Therefore, beyond 8 mins electrospinning time, which gave a coating with 13.7 *μ*m thickness in this study, the ultrasound attenuation evidently overpowered the beneficial effect of increased optical absorption.

**FIG. 4.**
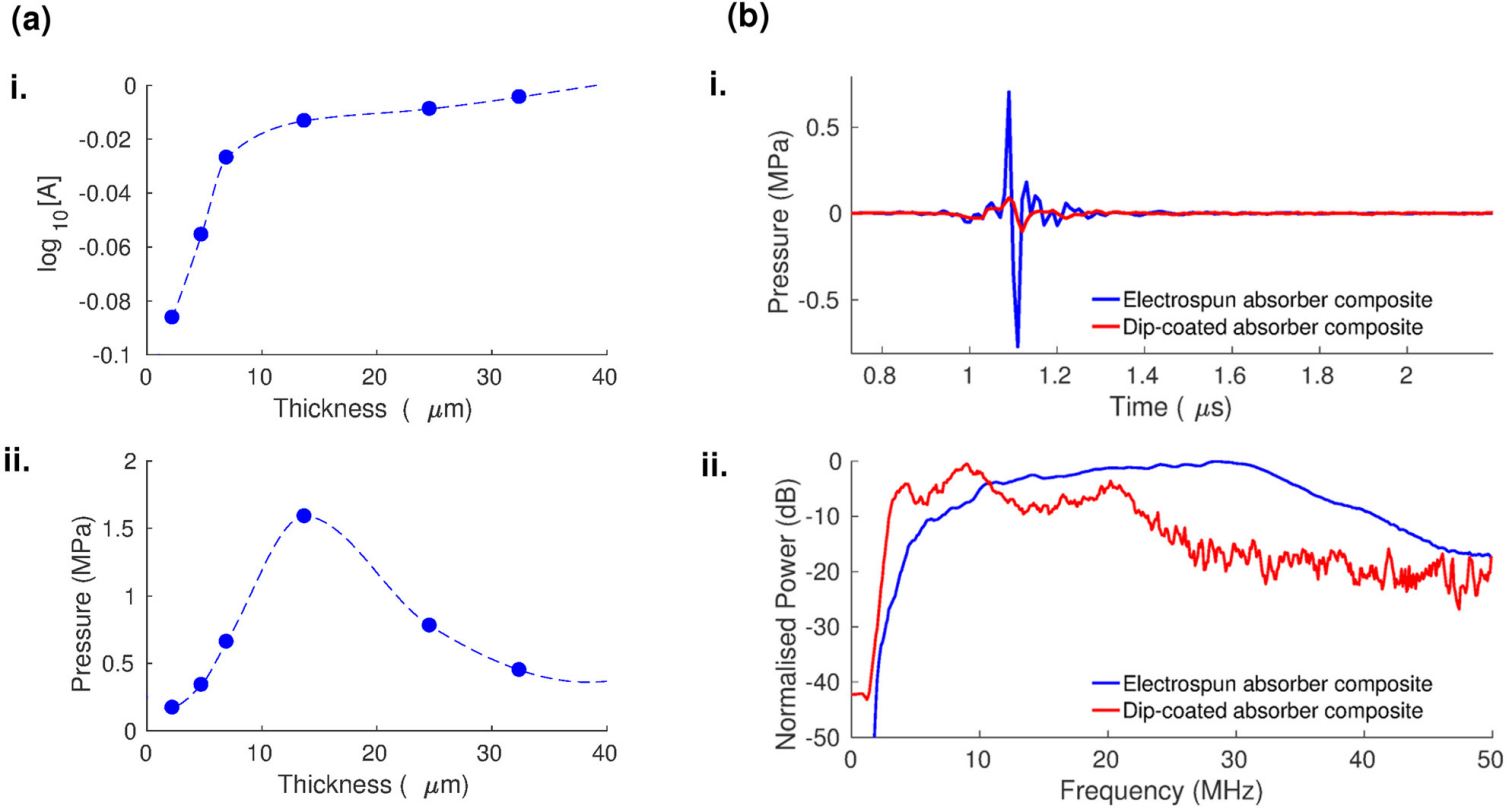
(a) Variation of the optical absorption [A] (i) and the generated ultrasound pressure (ii) with composite thickness for the electrospun multiwalled carbon nanotube (MWCNT)—polyvinyl alcohol (PVA) composite (dashed lines: interpolations between circular points). (b) Optical ultrasound pressures (i) and corresponding power spectra (ii) from fiber-optic transmitters prepared using electrospinning (blue) and dip coating (red) of the MWCNT-PVA composite.

For optical ultrasound transmitters, laser-induced damage threshold is a significant concern as it defines the maximum pulse energy available for pressure generation. Here, with a pulse energy of 11 *μ*J (fluence: 35 mJ/cm^2^) and a repetition rate of 100 Hz, the coating did not exhibit any noticeable degradation even after laser illumination for over 2 h. However, at pulse energies greater than 38 *μ*J (fluence: >0.12 J/cm^2^) at the same repetition rate, there was a pronounced decrease of around 30% in the measured ultrasound signal within seconds of illuminating the coating.

To better understand the dependence of the coating morphology on optical ultrasound generation capability, we evaluated the performance of our electrospun optical ultrasound transmitters against an equivalent reference formed by dip-coating. The reference transmitter was prepared by cleaving an optical fiber with core/cladding diameters of 200/220 *μ*m at normal incidence and dip-coated with the electrospinning precursor solution which was left to dry for 2 hours, followed by dip-coating in PDMS to obtain a solid film coating. For material equivalence in this comparison, the same corresponding polymer solutions (PVA-MWCNT polymer mixture and PDMS-toluene mixture in the electrospun absorber transmitter) were used to form the reference transmitter. The optical absorption of the dip-coated reference sample at 1064 nm was measured to be 0.97, identical to the optical absorption measured from the electrospun transmitter sample prepared with an electrospinning time of 8 minutes (thickness 13.7 *μ*m), to which it was compared. Although the optical absorption values were identical, the dip coated reference transmitter possessed a higher coating thickness (∼60 *μ*m) compared to its electrospun counterpart.

The fiber optic ultrasound transmitters with an electrospun absorber generated a fivefold greater ultrasound peak-to-peak pressure and a broader bandwidth relative to the reference dip-coated transmitter [Fig. 4(b)]. The measured peak-to-peak pressure was 1.59 MPa for the electrospun transmitter versus 0.31 MPa for the dip-coated reference [Fig. 4(b-i)]. The −6 dB bandwidth was measured as 29 MHz with the signal peak at 31 MHz for the electrospun transmitter compared to 8 MHz with the signal peak at 10.5 MHz for the reference [Fig. 4(b-ii)].

We attribute the improved ultrasound performance [Fig. 4(b)] of the electrospun transmitter compared to the dip-coated reference transmitter to two main factors: (i) lower ultrasound attenuation and (ii) improved opto-thermal energy conversion in the electrospun composite coating. Although both transmitters have exhibited identical optical absorption, the electrospun coating is much thinner than the dip-coated one. This is indicative of the electrospun coating possessing a higher absorption coefficient, which can be ascribed to the preferential alignment of the constituent MWCNT axis within and along the electrospun nanofibers [Fig. 2(d)] and consequently to the plane of polarization of the incident light from the multi-mode optical fiber end-face. As a consequence of the thinner coating, ultrasound attenuation is lower in the electrospun transmitter, resulting in higher ultrasound amplitude and wider bandwidth.^2^ Ultrasound attenuation in composites comprising Sylgard 184 PDMS (the elastomer used in our study) and carbonaceous nanoparticles has been found to be greater than that of pure Sylgard 184 PDMS,^13^ which indicates that all elements of our thermoelastic coating, especially the MWCNTs, contribute to the aforementioned ultrasound attenuation. Simultaneously, the high effective surface area of the porous electrospun absorber, and the complete elastomer infiltration through it, very likely promoted thermal energy transfer between these two constituents. These factors act synergistically to improve the opto-thermal energy conversion for optical ultrasound generation in the electrospun ultrasound transmitters.

The technique presented here could be further optimized in several ways. First, a focused electric field could be used to achieve better control over fiber deposition area, and a controlled mesh structure, to facilitate polarization selective absorption for multimodality imaging (for instance, a probe offering the functionalities of both ultrasound and photoacoustic imaging with polarization selective optical absorption). Second, the carrier polymer (PVA) could be carbonized from the optical fiber end-face prior to PDMS dip coating to obtain MWCNTs in planar alignment along the optical fiber tip to form the MWCNT-PDMS composite with minimal carrier polymer residue. Third, it may be possible to use PDMS itself as the electrospinning carrier polymer, which could enhance the elasticity of the coating and negate the need for dip-coating the elastomer. This would function to further reduce the number of fabrication steps and potentially improve the transmitter performance.

In conclusion, ultrasound generation was demonstrated on an optical fiber by electrospinning an absorber composite onto its end-face and then dip-coating with an elastomer. We were able to control the thickness of the coating layer by varying the electrospinning deposition time. High elastomer infiltration was observed by dip-coating PDMS elastomer into the porous nanofibrous absorber mesh produced. The electrospun transmitters produced ultrasound pressures in the MPa range with low pulse energies of 11 *μ*J, suitable for clinical use. Therefore, the feasibility of electrospinning directly on optical fiber tip and its applicability to form optical fiber ultrasound transmitters were illustrated in this study. In addition to probes for minimally invasive surgery, the paradigm of electrospinning on optical fiber end-faces could be of use in a range of applications including non-destructive testing of materials, chemical sensing, and environmental monitoring.^23,28,29^
